# Tracheal adenoid cystic carcinoma masquerading asthma: A case report

**DOI:** 10.1186/1471-2466-4-10

**Published:** 2004-10-19

**Authors:** Nurdan Kokturk, Sedat Demircan, Cuneyt Kurul, Haluk Turktas

**Affiliations:** 1 Gazi University School of Medicine, Department of Pulmonary Medicine, Ankara, Turkey; 2 Gazi University School of Medicine, Department of Thoracic Surgery, Ankara, Turkey

## Abstract

**Background:**

Tracheal tumors are often misdiagnosed as asthma and are treated with inhaled steroids and bronchodilators without resolution.

**Case Presentation:**

Here, a patient with tracheal adenoid cystic carcinoma who had been previously diagnosed with difficult asthma was reported. The possibility of the presence of localized airway obstruction was raised when the flow-volume curve suggesting fixed airway obstruction, was obtained.

**Conclusion:**

The presenting case report emphasizes the fact that not all wheezes are asthma. It is critical to bear in mind that if a patient does not respond to appropriate anti-asthma therapy, localized obstructions should be ruled out before establishing the diagnosis of asthma.

## Background

Tracheal tumors are uncommon and often overlooked until they reach to an advanced stage. The presenting symptoms are typically prolonged cough and wheezing that can be misdiagnosed as asthma [1]. Therefore, making precise diagnosis of tracheal tumor may be extremely challenging.

Here, a patient with tracheal adenoid cystic carcinoma who had been previously diagnosed with difficult asthma was reported.

## Case presentation

A 29 year-old man was referred to our hospital with a 2-year history of paroxysmal attacks of dyspnea, dry cough and wheezing. He had smoked 2 packs/day cigarettes for 3 years and has been ex-smoker for 5 years. He experienced frequent sudden-onset coughing episodes followed by the development of dyspnea and wheezing a year ago. He was previously diagnosed with difficult asthma and treated with high dose inhaled corticosteroids (1600 μg budesonide) and bronchodilators. Since he was unresponsive to the therapy, he has applied to several institutions for multiple times to seek medical attention.

**Figure 1:**
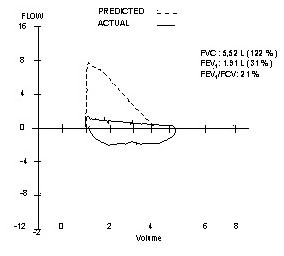
Flow-volume curve displays suggestive fixed airway obstruction

**Figure 2:**
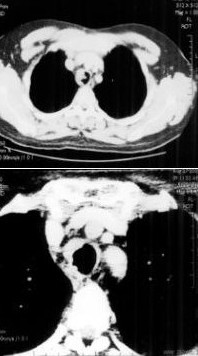
a) Chest CT scan displays a polypoid mass occupying 50 % of the lumen. b) Control CT scan displays resolution of the tumor

**Figure 3:**
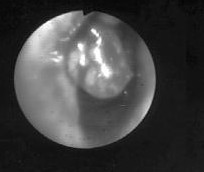
Bronchoscopic examination reveals polypoid mass originating from trachea with a 50 % obstruction of the lumen.

On admission, no stridor, wheezing and cyanosis were present and the general appearance was good. Vital signs were as follows: temperature 37°C, respiratory rate 20/min, pulse 96 beats/min, blood pressure 140/70 mmHg. The chest examination was unremarkable. The results of the routine laboratory analysis, including complete blood cell count, chemistry, arterial blood gas, urinalysis and chest x-ray were within normal ranges. On spirometric examination, flow-volume curve displayed suggestive fixed airway obstruction. Forced vital capacity (FVC) was 122 % of predicted, forced expiratory volume in one second (FEV1) was 31 % of predicted and FEV1/FVC was 21 % (Figure 1). In order to exclude the possibility of upper airway obstruction, a work-up of computerized tomography (CT) of the chest and fiberoptic bronchoscopy (FOB) was obtained. The CT scan illustrated a solid, polipoid intratracheal mass originating from the right side of the trachea at 4 centimeter proximal of the carina (Figure 2a). FOB revealed a smooth, round mass of 2 cm in diameter originating from the right lateral side of the trachea. The lesion was occupying approximately 50 % of the lumen (Figure 3). It localized at 4^th ^centimeter distal to larynx. Histopathological diagnosis was adenoid cystic carcinoma of the trachea.

The patient underwent resection surgery. At the operational site, there were severe adhesions between the mediastinal surface and the trachea. Therefore, a conservative surgery was performed. The tumor was seen on the right anterolateral wall of the trachea being expanded submucosally from the carina to the proximal end of the trachea.

The patient underwent adjuvant radiation therapy after the operation. CT scan of the neck revealed resolution of the tumor (Figure 2b). Now, 3 months after the operation, the patient has remained well.

## Conclusions

Primary tracheal tumors are rare with the incidence of less than 0.1 % [2,3]. The majority of tracheal tumors in adults are malignant and the most common ones are squamous cell carcinoma and adenoid cystic carcinoma (cylindroma). Tumors of the larynx and lungs are respectively, 75 and 180 times more common than malignant tracheal tumors [3,4]. Benign tracheal tumors such as lipoma, hamartoma and neurilemmoma are much more rare than malignant tracheal tumors [2,5,6].

Clinical manifestations of tracheal tumors are developed as a consequence of tumor bulk and location. Patients with tracheal tumor often have exertional shortness of breath, prolonged cough or a new onset of wheezing, which is frequently misdiagnosed as asthma. Patients are usually initially managed accordingly. However, tumor may occlude 75 % of the lumen before leading symptoms. In the literature, most of the reports highlight that there is always a remarkable delay of establishing accurate diagnosis as a result of misdiagnosis of asthma [1,5,7]. Pearson et al have reported a 2-month to 2-year delay in diagnosis in their series from Toronto General Hospital [8]. Therefore, adult-onset asthma that increases the severity despite the adequate therapy should alert one to the possibility of a central obstructing lesion [1]. In such patients, a flow-volume curve may provide extremely valuable data and may lead the clinician toward accurate diagnosis.

Another diagnostic challenge of tracheal tumor is the fact that it can occasionally be visualized by plain chest X-ray. CT scan of the chest or magnetic resonance imaging may yield more valuable data on the site and length of the tracheal lesion [1]. The cornerstone diagnostic modality is bronchoscopy [1].

In the presenting report, the patient had been mistakenly diagnosed with difficult asthma because of the presence of uncontrolled asthmatic symptoms and poor lung functions despite the use of high doses corticosteroids. Nevertheless, the flow-volume curve typically displayed localized fixed obstruction of central airways. This led us to have a work-up of CT scan and FOB to rule out upper airway obstructions.

Adenoid cystic carcinomas are smooth, firm, and well-circumscribed lesions. These tumors grow extremely slowly. Patients have been known to survive for 10 to 15 years with multiple lung metastases. Spread tends to occur submucosally [1].

The optimal therapeutic approach is surgical resection and reconstruction. The surgeon should be aware of the fact that the apparent gross margin of the tumor is usually still involved with the tumor cells so that the resection should be done at least 1 cm beyond the gross tumor margin. Postoperative irradiation was recommended by most of the authors [1,9]. Long-term postoperative follow-up is important to discover recurrences.

In this case, postoperative irradiation on curative doses has been applied for a month. Control bronchoscopic examination revealed near-complete remission.

The presenting case report emphasizes the fact that not all wheezes are asthma. It is critical to bear in mind that if a patient does not respond to appropriate anti-asthma therapy, localized airway obstructions should be rule out before establishing the diagnosis of asthma.

## Competing interest

The author(s) declare that they have no competing interests.

## Authors' contributions

HT has seen the patient and made the diagnosis of tracheal obstruction, participated in the design of the manuscript. NK has seen the patient in the clinical, followed the patient and drafted the manuscript. SD and CK have performed the surgery and followed the patient. All authors read and approved the final manuscript.

## Pre-publication history

The pre-publication history for this paper can be accessed here:



## References

[B1] Allen MS (1993). Malignant Tracheal Tumors.. Mayo Clin Proc.

[B2] Tastepe AI, Kuzucu A, Demircan S, Liman TS, Demirag F (1998). Surgical Treatment of Tracheal Hamartoma.. Scand Cardiovasc J.

[B3] Pando Pinto JM, Vega Cuadri A, Montero Garcia C, Blasco Huelva A (2000). Primary Carcinoma Of The Trachea. Report of 2 Cases.. An Otorrinolaringol Ibero Am.

[B4] Stack PS, Steckler RM (1990). Tracheal Neurilemmoma: Case Report and Review Of The Literature.. Head And Neck.

[B5] Turay UY, Ergun P, Topcu S, Kurul C, Aydogdu M, Demirag F, Erdogan Y (2002). A Case Of Tracheal Neurilemmoma Treated As Bronchial Asthma.. Turkish Respir J.

[B6] Tayama K, Takal E, Ueda T, Yano T, Ichinose Y (1996). Tracheal Lipoma Obstructing The Right Main Bronchus: Report Of A Case.. Surg Today.

[B7] Parrish RW, Banks J, Fennerty AG (1983). Tracheal Obstruction Presenting As Asthma.. Postgrad Med J.

[B8] Pearson FG, Todd TRJ, Cooper JD (1984). Experience With Primary Neoplasms Of The Trachea And Carina.. J Thorac Cardiovasc Surg.

[B9] Grillo HC, Mathisen DJ (1990). Primary Tracheal Tumors: Treatment and Results.. Ann Thorac Surg.

